# Isolation of ferret astrocytes reveals their morphological, transcriptional, and functional differences from mouse astrocytes

**DOI:** 10.3389/fncel.2022.877131

**Published:** 2022-10-06

**Authors:** Jureepon Roboon, Tsuyoshi Hattori, Dinh Thi Nguyen, Hiroshi Ishii, Mika Takarada-Iemata, Takayuki Kannon, Kazuyoshi Hosomichi, Takashi Maejima, Kengo Saito, Yohei Shinmyo, Michihiro Mieda, Atsushi Tajima, Hiroshi Kawasaki, Osamu Hori

**Affiliations:** ^1^Department of Neuroanatomy, Graduate School of Medical Sciences, Kanazawa University, Kanazawa, Japan; ^2^Department of Bioinformatics and Genomics, Graduate School of Advanced Preventive Medical Sciences, Kanazawa University, Kanazawa, Japan; ^3^Department of Integrative Neurophysiology, Graduate School of Medical Sciences, Kanazawa University, Kanazawa, Japan; ^4^Department of Medical Neuroscience, Graduate School of Medical Sciences, Kanazawa University, Kanazawa, Japan

**Keywords:** neocortex, astroglia, primary culture, higher mammals, cell proliferation, morphology, calcium, brain evolution

## Abstract

Astrocytes play key roles in supporting the central nervous system structure, regulating synaptic functions, and maintaining brain homeostasis. The number of astrocytes in the cerebrum has markedly increased through evolution. However, the manner by which astrocytes change their features during evolution remains unknown. Compared with the rodent brain, the brain of the ferret, a carnivorous animal, has a folded cerebral cortex and higher white to gray matter ratio, which are common features of the human brain. To further clarify the features of ferret astrocytes, we isolated astrocytes from ferret neonatal brains, cultured these cells, and compared their morphology, gene expression, calcium response, and proliferating ability with those of mouse astrocytes. The morphology of cultured ferret astrocytes differed from that of mouse astrocytes. Ferret astrocytes had longer and more branched processes, smaller cell bodies, and different calcium responses to glutamate, as well as had a greater ability to proliferate, compared to mouse astrocytes. RNA sequencing analysis revealed novel ferret astrocyte-specific genes, including several genes that were the same as those in humans. Astrocytes in the ferret brains had larger cell size, longer primary processes in larger numbers, and a higher proliferation rate compared to mouse astrocytes. Our study shows that cultured ferret astrocytes have different features from rodent astrocytes and similar features to human astrocytes, suggesting that they are useful in studying the roles of astrocytes in brain evolution and cognitive functions in higher animals.

## Introduction

Astrocytes are one of the three major glial cell types in the brain and play multiple roles by supporting the central nervous system structure (Schiweck et al., 2018), providing neurotrophic factors (Schwartz and Nishiyama, 1994; Pöyhönen et al., 2019), facilitating the formation of the blood-brain barrier (Janzer and Raff, 1987; Abbott et al., 2006), regulating synapse formation (Bolton and Eroglu, 2009; Chung et al., 2015), and maintaining central nervous system homeostasis (Dani et al., 1992; Gee and Keller, 2005). Astrocytes affect cognitive processing, including learning, memory, and emotionality by regulating the connectivity of neural networks (Orr et al., 2015; Nagai et al., 2019). Astrocyte dysfunction is associated with neurodevelopmental diseases such as tuberous sclerosis complex, Rett syndrome, fragile X syndrome, and autism spectrum disorder (McGann et al., 2012; Molofsky et al., 2012). Interestingly, a previous study showed that mice engrafted with human astrocytes exhibited improved learning, suggesting that astrocytes contribute to improving brain function (Han et al., 2013).

Crucial advances in the mammalian brain during evolution include the expansion and folding of the cerebral cortex. Moreover, the relative number of astrocytes to neurons in the human cortex is higher than that in the rodent cortex (Nedergaard et al., 2003). Human astrocytes have much larger diameters, more complex structures, and longer processes compared to rodent astrocytes (Colombo, 1996; Oberheim et al., 2009). Although rodent astrocytes respond to both ATP and glutamate by increasing their intracellular calcium concentrations, fetal human astrocytes do not respond to glutamate but respond to ATP (Zhang et al., 2016). The propagation of calcium waves is faster in human astrocytes than in rodent astrocytes (Oberheim et al., 2009; Han et al., 2013). These studies suggest that the properties of astrocytes were altered during evolution, which may contribute to the brain structure and function in higher mammals. However, the molecular and cellular mechanisms underlying changes in astrocytes and their contribution to cerebral cortex evolution remain unclear.

Compared with the rodent brain, the ferret brain has a folded cerebral cortex, higher white to gray matter ratio, and ventral hippocampus (Barnette et al., 2009). Therefore, ferrets have been widely used to investigate the mechanisms underlying the development and evolution of well-developed brains (Gilardi and Kalebic, 2021). Ferret astrocytes in the visual cortex have more territory volume and overlap with neighboring astrocyte processes compared to mouse astrocytes (López-Hidalgo et al., 2016; Zhang et al., 2016). Visually evoked calcium responses in ferret visual cortex astrocytes are robust and highly tuned to visual stimuli, whereas visual responses in mouse astrocytes are weak (Schummers et al., 2008). These studies indicate that investigating ferret astrocytes may facilitate an understanding of their role in higher brain functions.

In this study, to clarify the features of ferret astrocytes, we for the first time isolated and cultured astrocytes from neonatal ferret brains. Our results show that cultured ferret astrocytes have different cell morphology, gene expression, calcium response, and proliferation ability from rodent astrocytes and features similar to human astrocytes. Therefore, ferret astrocytes may be a useful tool for studying the roles of astrocytes in brain evolution and higher cognitive functions.

## Materials and methods

### Animals

Normally pigmented sable ferrets (*Mustela putorius furo*) were purchased from Marshall Farms (North Rose, NY, USA) and maintained as previously described (Kawasaki et al., 2004; Iwai and Kawasaki, 2009). Wild-type ICR mice were maintained as previously described (Roboon et al., 2021). The day of birth was defined as postnatal day 0 (P0). All procedures were performed in accordance with protocols approved by the Animal Care and Use Committee of Kanazawa University (AP-183919).

### Astrocyte primary culture

Astrocyte cultures were prepared from the cerebral cortices of postnatal day 1 (P1) to P3 neonatal mice and P1 ferrets following the McCarthy and de Vellis (1980) astrocyte model. Briefly, cerebral cortices were digested at 37°C in Hanks’ Balanced Salt Solution containing 2 mg/ml dispase II (383-02281, Wako, Osaka, Japan). The cells were plated in poly D-lysine-coated flasks (356537, Corning, Inc., Corning, NY, USA) and cultured in Dulbecco’s modified Eagle’s medium (044-29765, Wako) supplemented with 10% fetal bovine serum. After 14 days of cultivation, the culture flasks were shaken at 200 rpm for 12 h and the supernatant was discarded. Astrocytes were detached using 0.05% trypsin-EDTA (15400054, Thermo Fisher Scientific, Waltham, MA, USA), plated at a density of 1 × 10^5^ cells/cm^2^ into culture dishes, and cultured in the media for glial cells described above. For cryopreservation, astrocytes were frozen in 10% dimethyl sulfoxide (1340655, Nacalai Tesque, Kyoto, Japan) in astrocyte culture medium for 24 h and then transferred to a liquid nitrogen storage tank.

### RNA sequencing transcriptome profiling

Astrocytes were cultured in the culture media containing 10% fetal bovine serum for 3 days after plating, after which the media were replaced with serum-free growth media [50% Neurobasal Medium (21103049, Thermo Fisher Scientific), 50% Dulbecco’s modified Eagle’s medium, 100 U/ml of penicillin, 100 μg/ml of streptomycin, 1 mm of sodium pyruvate, 2 mm of L-glutamine, 1 μg/ml of transferrin (T1147, Sigma-Aldrich, St. Louis, MO, USA), 1 μg/ml of bovine serum albumin, 0.16 μg/ml of putrescine (P5780, Sigma-Aldrich), 0.6 ng/ml of progesterone (P8783, Sigma-Aldrich), 0.4 ng/ml of sodium selenite (S5261, Sigma-Aldrich), 5 μg/ml of N-Acetyl Cysteine (NAC), and 5 ng/ml of heparin-binding EGF-like growth factor (E4643, Sigma-Aldrich)]. Total RNA from cultured ferrets and mouse astrocytes was harvested on day 7 and used for RNA library preparation using the TruSeq Stranded mRNA Sample Preparation Kit (20020594, Illumina, San Diego, CA, USA), with polyA selection for ribosomal RNA depletion. Libraries were generated in duplicate using 500 ng of total RNA extracted from cultured ferrets or mouse astrocytes. The libraries were sequenced using an Illumina HiSeq 2000 to obtain paired-end 101-bp reads for each sample.

RNA-sequencing (RNA-seq) reads were aligned to their reference genomes using STAR v2.7.0f (Dobin et al., 2013) with default settings. The following Ensembl reference genomes were used: ferret, MusPutFur1.0.99; mouse, GRCm38.99 (Cunningham et al., 2019). Gene expression profiles were quantified using Cufflinks v2.2.1 (Trapnell et al., 2012) from the aligned RNA-seq reads with gene annotation information corresponding to the reference genomes (Liao et al., 2014). Gene expression levels are represented as fragments per kilobase of exon per million reads mapped (FPKM) and normalized by the number of RNA fragments mapped to the reference genome and total length of all exons in the transcript. Read counts from the RNA mapping were obtained by “featureCounts” (Liao et al., 2014). Read counts, FPKM and gene length of all the transcripts were listed in the Supplementary Table 1.

### Protein extraction and western blot analyses

Astrocytes cultured in media containing serum for 4 days were lysed in RIPA lysis buffer containing 1% NP-40, 0.1% sodium dodecyl sulfate, 0.2% deoxycholate, and protease inhibitors (1 mm phenylmethylsulfonyl fluoride and 1 μg/ml aprotinin), and total protein was extracted. Equal concentrations of denatured protein lysates were separated by sodium dodecyl sulfate-polyacrylamide gel electrophoresis and transferred onto polyvinylidene fluoride membranes. The membranes were blocked with 5% skimmed milk for 30 min and incubated with primary antibodies against GFAP (1:2000; G9269, Sigma), vimentin (1:500, SC-7557, Santa Cruz Biotechnology, Dallas, TX, USA), NDRG2 (1:1000, SC-19468, Santa Cruz Biotechnology), EAAT1 (1:1000, DS130-095-814-5-21-2, Miltenyi Biotec, Gladbach Bergisch, Germany), SOX9 (1:1000, af3075, R&D Systems, Minneapolis, MN, USA), PMP2 (1:1000, 12717-1-AP, Proteintech, Rosemont, IL, USA), CALCOCO2 (1:1000, 12229-1-AP, Proteintech), and β-actin (1:2000, 281-98721, FUJIFILM Wako Chemicals, Osaka, Japan) at 4°C for 16 h. The membranes were washed three times with Tris-buffered saline containing Tween 20 and incubated with anti-rabbit (1:5000, sc-2004, Santa Cruz Biotechnology), anti-goat (1:1000, sc-2354, Santa Cruz Biotechnology), or anti-mouse (1:5000, sc-516102, Santa Cruz Biotechnology) secondary antibodies at room temperature for 2 h. Immunoreactivity was detected using an enhanced chemiluminescence system (Luminata Forte HRP substrate, 61-0196-09, Merck Millipore, Billerica, MA, USA). The results were quantified using ImageJ version 1.52p software (NIH, Bethesda, MD, USA)^1^.

### Calcium imaging

Astrocytes cultured in serum-containing media for 4 days were loaded with a calcium indicator, 2.5 μm Fura-2AM (Dojindo Laboratories, Kumamoto, Japan), and incubated at 37°C for 40 min. After 15 min of incubation in the culture media without Fura-2AM, the coverslip containing the cells was transferred to an imaging chamber with an external solution (140 mm NaCl, 2.5 mm KCl, 2 mm CaCl_2_, 1 mm MgCl_2_, 10 mm HEPES, and 10 mm glucose, pH 7.4, adjusted with NaOH) flowing at 1 ml/min at room temperature. Fluorescence images were acquired using an upright microscope (BX51WI, Olympus, Tokyo, Japan) equipped with a water immersion objective (×40, numerical aperture 0.8; Olympus), mercury light source, filter wheel system (ProScan II, Prior Scientific, Rockland, MA, USA), and a cooled CCD camera (CoolSNAP HQ2, Photometrics, Tucson, TZ, USA). A set of two images at 340 and 380 nm emission frequencies was recorded every 5 s through a dichroic mirror (415 nm) and an emission filter (500–530 nm) using imaging software (MetaFluor, Molecular Devices, Sunnyvale, CA, USA). Fluorescence signals from selected regions of interest were background-corrected and expressed as the ratio of fluorescence intensities at 340 and 380 nm.

### 5-Bromo-2′-deoxyuridine labeling

The proliferation of astrocytes cultured in media containing serum was evaluated through double labeling with 5-bromo-2-deoxyuridine (BrdU, 05650-66, Nacalai Tesque) and GFAP-positive cells on day 7. The cells were incubated with BrdU (10 μm) in culture medium for 24 h, fixed with 4% paraformaldehyde (PFA), and permeabilized with 0.1% Triton-X100. BrdU epitope was exposed by incubating the cells in 2 M hydrochloric acid at 37°C for 1 h, followed by neutralization with 0.1 M sodium borate (pH 8.5) for 20 min. The cells were then examined using immunocytochemistry, as described below.

### Immunocytochemistry

Cells cultured in serum-containing media were fixed with 4% PFA for 10 min and permeabilized with 0.1% Triton X-100 on day 7. The cells were blocked with 3% bovine serum albumin and 0.3% Triton-X100 in phosphate-buffered saline (PBS) for 30 min. The cells were stained with antibodies against GFAP (1:1000), MBP (1:500, MAB386, Merck Millipore), Iba1 (1:300, 019-19741, Wako), NeuN (1:300, MAB377, Merck Millipore), β-III tubulin (1:500, MAB1637, Merck Millipore), BrdU (1:25, B44, BD Biosciences, Franklin Lakes, NJ, USA), and Ki67 (1:300, bs-2130, Bioss, Woburn, MA, USA). Immunocytochemical labeling was visualized with Alexa Fluor™ 488 (1:200, Thermo Fisher Scientific) or Cy3 (1:200, Jackson ImmunoResearch Laboratories, West Grove, PA, USA)-conjugated secondary antibodies. Cell nuclei were visualized using DAPI (D1306, Molecular Probes, Eugene, OR, USA).

### Immunohistochemistry

The animals were deeply anesthetized with an anesthetic mixture containing medetomidine, butorphanol, and midazolam and subjected to transcardial perfusion with PBS, followed by 4% PFA. The brains were post-fixed in 4% PFA overnight and dehydrated in 30% sucrose for 72 h. The brains were embedded in OCT compound and stored at −80°C. Free-floating coronal sections were prepared using a cryostat. The sections were washed with PBST (0.3% Triton X-100 in PBS), blocked with 3% bovine serum albumin and 0.3% Triton X-100 in PBS for 30 min, and incubated overnight with antibodies against GFAP (1:1000), S100β (1:300, S2532, Sigma-Aldrich), Ki67 (1:100) and GFP (1:500, 04404-84, Nacalai Tesque). The sections were washed and labeled with secondary antibodies conjugated to Alexa Fluor™ 488 (1:200) or Cy3 (1:200). Cell nuclei were visualized using DAPI.

### *In utero* electroporation for ferrets and mice

*In utero* electroporation for ferrets was performed as described previously (Kawasaki et al., 2012; Shinmyo et al., 2017). Briefly, the uterine horns of anesthetized pregnant ferrets at E31 were exposed and kept wet by adding drops of PBS intermittently. The location of embryos was visualized with transmitted light delivered through an optical fiber cable. Approximately 2 to 5 μl of DNA solution of pCAG-PBase and pPB-CAG-EGFP described previously (Hamabe-Horiike et al., 2021) were injected into the lateral ventricle using a pulled glass micropipette. Each embryo within the uterus was placed between tweezer-type electrodes with a diameter of 5 mm (CUY650-P5; NEPA Gene, Japan). Square electric pulses (100 V, 50 ms) were passed five times at 1-s intervals using an electroporator (ECM830, BTX, Cambridge, UK). The wall and skin of the abdominal cavity were sutured, and the embryos were allowed to develop normally. *In utero* electroporation for mice was performed as described previously (Wakimoto et al., 2015). The same DNA solution described above was injected into the lateral ventricle at E15.

### Image analysis

For *in vitro* studies, three to five independent cultures were established, and cells in multiple fields were counted for each culture. For *in vivo* studies, three to four brain sections from each animal were analyzed. Each field was imaged using z-stacks with 2 μm steps consisting of ten planes. To evaluate astrocyte proliferation, we analyzed the ratio of Ki67/GFAP or BrdU/GFAP double-positive to GFAP-positive cells. Astrocyte proliferation was analyzed in the corpus callosum and layers 2–6 of the cortex. Images were captured using a BZ-x710 microscope (Keyence, Osaka, Japan), and the cells were counted using ImageJ software. Morphology of astrocytes was analyzed in layer 2–6 of the cortex. Fluorescence images (1 μm intervals) were acquired with a laser confocal microscope (Dragonfly; Andor, Belfast, Northern Ireland) equipped with an water-immersion objective lens (×60). Stacked images were obtained using the Fiji package of ImageJ.

### Statistical analysis

The results are expressed as the mean ± standard error of the mean (SEM), with the number of experiments indicated by *n*. An unpaired two-tailed Student’s *t*-test was used to evaluate the statistical significance of the results. Differences were considered statistically significant at *P* < 0.05.

## Results

### Isolation of ferret astrocytes

**FIGURE 1:**
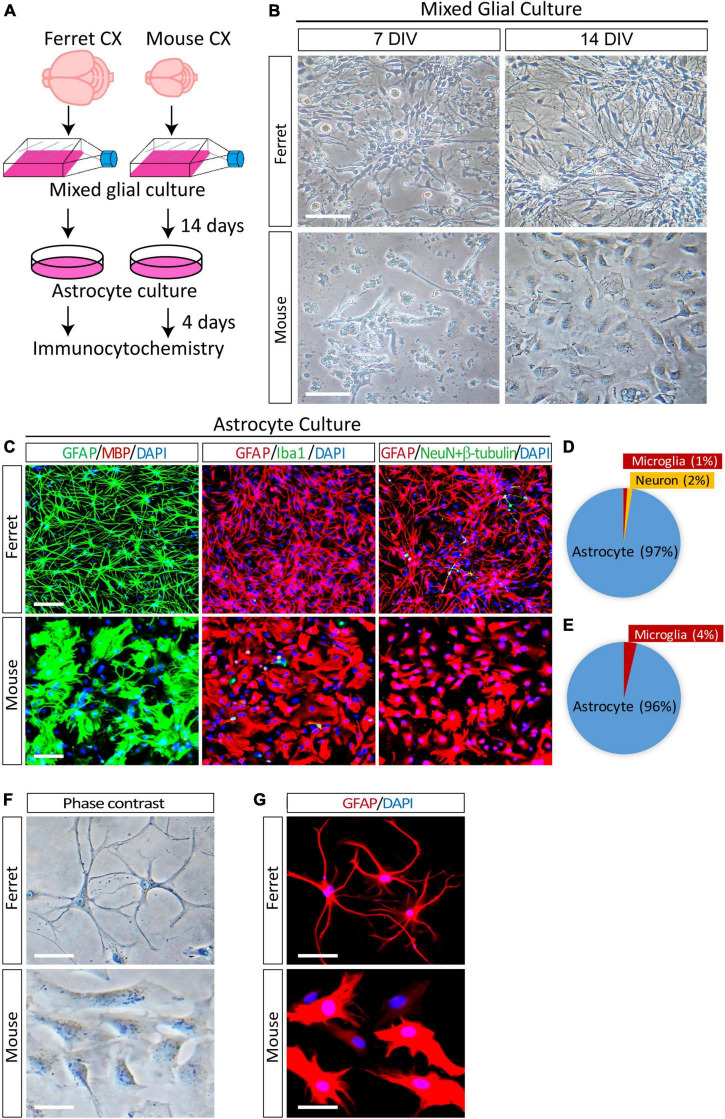
Purification and morphological analysis of ferret and mouse astrocytes. **(A)** Experimental procedure used to culture ferret and mouse astrocytes. Mixed glial cells and astrocytes were cultured in media containing 10% fetal bovine serum. **(B)** Representative phase-contrast images of ferret and mouse mixed glial culture at 7 and 14 days *in vitro* (DIV). Scale bars = 100 μm. **(C)** Cultured ferret and mouse astrocytes were subjected to immunocytochemistry of GFAP, MBP, Iba1, NeuN, and β-III tubulin at 4 days after plating. Nuclei were counterstained with DAPI. Scale bars = 100 μm. **(D,E)** Proportion of astrocytes, oligodendrocytes, microglia, and neurons in ferret and mouse astrocyte cultures. *n* = 3 independent cultures. **(F,G)** Representative images of phase-contrast and immunocytochemistry of ferret and mouse astrocytes at 4 days after plating, with GFAP antibody. Nuclei were counterstained with DAPI. Scale bars = 20 μm.

In the ferret brain, astrogenesis begins during the last 4 days of embryonic development (E38–E41) and continues for 2 weeks in the postnatal brain (Gilardi and Kalebic, 2021). In mice, astrogenesis starts at approximately E18 and continues for at least a week after birth (Reemst et al., 2016). To obtain proliferating astrocyte cultures, we isolated mixed glial cells from the cortices of neonatal P1 ferrets and P1–P3 mice as described previously (McCarthy and de Vellis, 1980). To achieve the proper astrocyte density, we cultured glial cells from one ferret pup in four T75 tissue culture flasks. In contrast, we used glial cells from the cortices of four mouse pups in a single T75 flask. The mixed glial cells were cultured in an astrocyte culture medium supplemented with 10% fetal bovine serum for 14 days (Figure 1A). On day 7, ferret cells grew faster than mouse cells and covered most of the surface area of the culture flasks (Figure 1B). After 14 days of growth, the flasks were shaken overnight, and the supernatant was discarded to remove microglia and oligodendrocytes from the mixed glial culture. The remaining astrocytes were detached using trypsin-EDTA, replated on culture dishes, and cultured in media containing serum. To examine the purity of the astrocyte cultures at 4 days after plating, the cells were immunostained with antibodies against cell type-specific markers of astrocytes (GFAP), oligodendrocytes (MBP), microglia (Iba1), and neurons (NeuN and βIII-tubulin). The ferret astrocyte culture contained 97% astrocytes, 1% microglia, and 2% neurons (Figures 1C,D). In contrast, the mouse astrocyte culture contained 96% astrocytes and 4% microglia (Figures 1C,E). In addition, the purity of replated ferret astrocytes after freezing in liquid nitrogen and thawing was 100% (Supplementary Figure 1).

Next, to examine the morphological differences between cultured astrocytes from ferret and mouse, we performed phase contrast observation and immunocytochemistry using an anti-GFAP antibody. There were more branched and longer processes and smaller cell bodies in ferret astrocytes than in mouse astrocytes (Figures 1F,G). Furthermore, although mouse astrocytes showed a cobblestone-like pattern and closely adhered to adjacent cells, ferret astrocytes did not adhere to each other, even after they grew and covered most of the culture dish surface.

### Comparison of ferret and mouse astrocyte transcriptomes

The gene expression profiles of cultured ferret and mouse astrocytes were compared using RNA-seq. To reduce the effect of serum in the culture media on gene expression (Foo et al., 2011), total RNA was prepared from astrocytes cultured in serum-free media for 4 days (days 3–7). We calculated FPKM of each gene (Supplementary Table 1) and identified ferret- or mouse-specific astrocyte genes (FPKM <1 in non-enriched species, ranked by FPKM in enriched species; Table 1). A comparison of the results with those of a previous study using human and mouse astrocytes (Zhang et al., 2016) revealed that several of the ferret astrocyte-specific genes were also present in humans, including *Calcoco2* and *Pmp2* (Table 2). We further confirmed that PMP2 and CALCOCO2 protein expression were significantly higher in ferret astrocytes than in mouse astrocytes (Supplementary Figure 2).

**TABLE 1:** Expression of top 20 species-specific genes.

Genes in ferret astrocytes not found in mouse astrocytes			Genes in mouse astrocytes not found in ferret astrocytes		
Gene symbol	Ferret astrocyte expression	Mouse astrocyte expression	Gene symbol	Ferret astrocyte expression	Mouse astrocyte expression
*Calcoco2*	180.2	0.02	*Serpinf1*	0.11	641.5
*Ptp4a1*	144.6	0.58	*Lcn2*	0.00	460.7
*Cyp26a1*	140.9	0.02	*Tmem176b*	0.06	201.5
*Aldh1a3*	115.8	0.30	*Cavin2*	0.34	179.0
*Agt*	108.6	0.71	*Aldh1a1*	0.63	153.9
*Psmd5*	102.6	0.63	*Tyrobp*	0.69	145.5
*Rtl5*	101.7	0.67	*Cyp26b1*	0.35	144.9
*Pam*	86.6	0.00	*Cemip*	0.21	123.0
*Mpz*	84.7	0.06	*Tmem176a*	0.00	121.9
*Pmp2*	78.2	0.09	*C1qb*	0.57	119.2
*Pygm*	69.6	0.46	*Ctss*	0.68	105.9
*Ldoc1*	51.2	0.03	*Pcolce*	0.71	90.7
*Nts*	49.4	0.00	*Mpeg1*	0.45	84.0
*St6galnac2*	48.0	0.28	*Mgp*	0.00	74.4
*Ifi44l*	47.9	0.06	*Mfsd2a*	0.76	66.9
*Dut*	41.4	0.00	*Thbd*	0.27	64.1
*Pdlim3*	39.0	0.12	*C3*	0.06	60.4
*Ppp2r2c*	35.1	0.56	*Apobec1*	0.00	58.7
*Gpr83*	33.0	0.08	*Slc39a12*	0.42	54.6
*Sbspon*	31.1	0.13	*Laptm5*	0.20	50.5

Genes were ranked by FPKM. Species-specific astrocyte genes have FPKM values <1 in astrocytes from non-enriched species.

**TABLE 2:** Human-astrocyte specific genes identified in ferret astrocytes (Zhang et al., 2016).

Genes in human and ferret astrocytes not found in mouse astrocytes		
Gene symbol	Ferret astrocyte expression	Mouse astrocyte expression
*Calcoco2*	157.1	0.03
*Pmp2*	51.5	0.08

Top 20 Ferret-enriched genes were compared with top 20 human-enriched genes (Zhang et al., 2016). Genes in human and ferret astrocytes not found in mouse astrocytes were ranked by FPKM.

Next, we compared the expression of astrocyte-specific genes (Boisvert et al., 2018) in the cells treated with serum-free media. Astrocyte-markers were expressed in both species, although the expression levels of *Aqp4*, *Aldh1l1*, *Apoe*, *Ndrg2*, *Slc1a2*, and *Slcla3* were lower in ferret astrocytes (Table 3). In contrast, *S100b* and *Aldoc* were higher in ferret astrocytes.

**TABLE 3:** Expression levels of astrocyte-specific marker genes (FPKM) in ferret and mouse astrocytes.

Astrocyte-specific markers		
Gene symbol	Ferret astrocyte expression	Mouse astrocyte expression
*Gfap*	1,501.6	1,487.1
*Vim*	1,229.8	4,261.9
*Aqp4*	23.2	205.7
*S100b*	245.5	60.2
*Aldh1l1*	9.8	82.7
*Apoe*	12.0	3,174.9
*Aldoc*	1,659.6	965.4
*Sox9*	17.6	33.5
*Ndgr2*	20.8	161.2
*Slc1a2*	0.1	49.4
*Slc1a3*	80.5	180.8

### Expression of astrocyte markers in cultured ferret astrocytes

**FIGURE 2:**
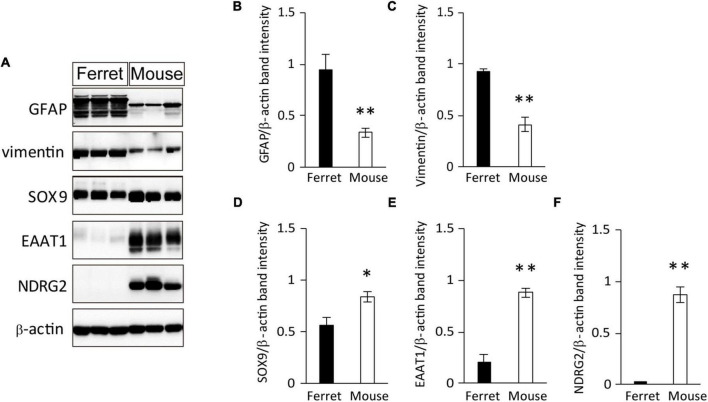
Expression of astrocyte-specific markers. **(A)** Ferret and mouse astrocytes cultured with serum for 4 days were subjected to western blot analysis of astrocyte-specific markers GFAP, vimentin, SOX9, EAAT1, NDRG2, and β-actin. **(B–F)** Relative optical density of astrocyte-specific markers normalized to the loading control β-actin. *n* = 5 independent cultures. Data represent the means ± SEM. The *P*-values were determined using paired Student’s *t*-test. *P < 0.05 and ^**^P < 0.01.

To further investigate the protein expression of astrocyte markers in cultured ferret astrocytes, we performed western blot analysis using antibodies against GFAP, vimentin, SOX9, EAAT1, and NDRG2. All of these markers, except for NDRG2, were expressed in ferret astrocytes (Figures 2A–F). The expression levels of GFAP were significantly higher than those of the other markers (Figures 2A–C), whereas the SOX9 and EAAT1 expression levels were significantly lower in ferret astrocytes than in mouse astrocytes (Figures 2A,D,E). These results indicate that markers of rodent astrocytes, such as GFAP, vimentin, and SOX9, can also be used for ferret astrocytes.

### Calcium response of cultured ferret astrocytes

**FIGURE 3:**
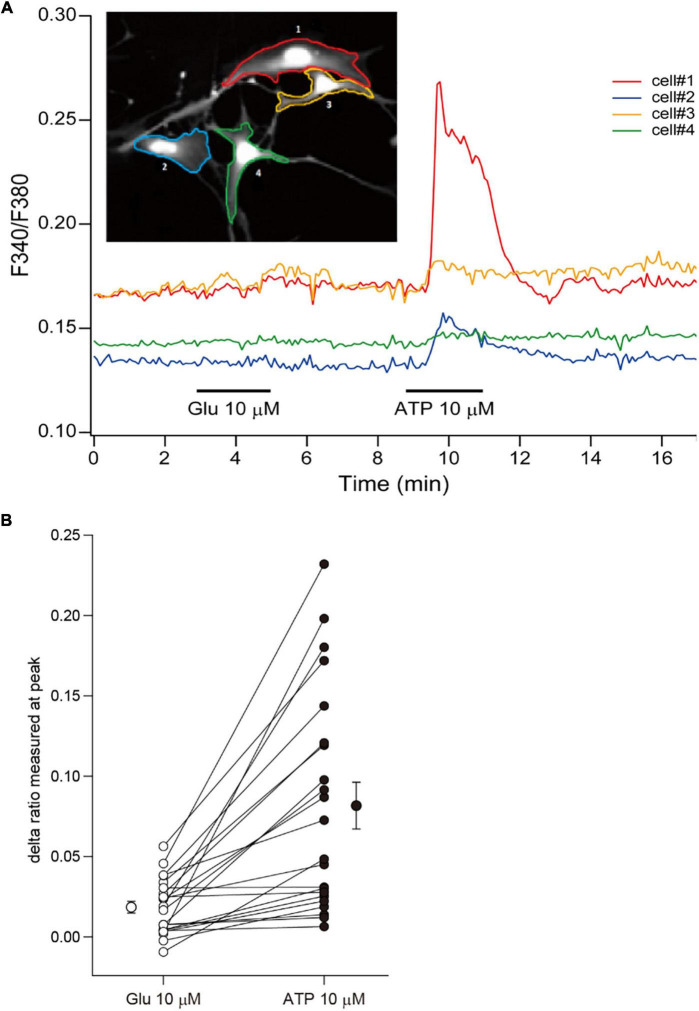
Calcium responses of ferret astrocytes. **(A)** Effect of bath-applied glutamate (10 μm) and ATP (10 μm) on intracellular calcium concentration [(Ca^2+^)_*i*_] of ferret astrocytes. The [Ca^2+^]_i_ was measured from the fluorescent images of cells and expressed as the ratio of Fura-2 intensities at 340 and 380 nm (F340/F380). The time course of changes in the F340/F380 of individual cells shown in the inset are plotted by the corresponding-colored lines depicting the regions of interest. **(B)** Summary plots representing the changes in Fura-2 ratio following treatments of individual cells with glutamate and ATP, expressed as delta ratio (peak ratio during reagent application minus ratio prior to application). The mean values (±SEM, *n* = 22 cells from 1 independent culture) of the delta ratios induced by each reagent are plotted beside the individual data.

Although rodent astrocytes respond to sensory input, synaptic glutamate, and extracellular ATP by increasing their intracellular calcium concentrations, cultured fetal human astrocytes, which proliferate robustly, respond to ATP but not to glutamate (Zhang et al., 2016). In contrast, cultured adult human astrocytes that do not divide respond to both ATP and glutamate. To investigate the calcium response in ferret astrocytes, we performed calcium imaging experiments using Fura-2AM. Cultured ferret astrocytes clearly responded to ATP by increasing their increasing calcium levels but did not respond to glutamate (Figures 3A,B). Therefore, the calcium response properties are similar between cultured ferret neonatal and human fetal astrocytes.

### Proliferation and morphology of ferret astrocytes

**FIGURE 4:**
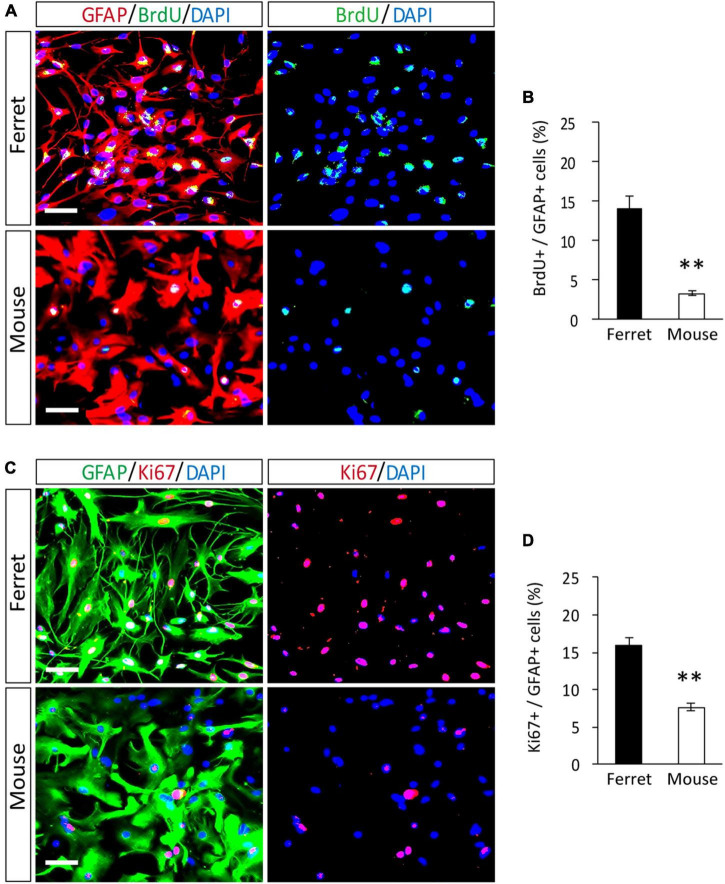
Proliferation of ferret and mouse astrocytes. **(A–D)** Ferret and mouse astrocytes cultured with serum for 4 days were subjected to immunocytochemistry with antibodies against GFAP, BrdU, and Ki67. Nuclei were counterstained with DAPI. Scale bars = 50 μm. **(B,D)** Proportion of BrdU- or Ki67-positive cells in GFAP-positive astrocytes. *n* = 5 independent cultures. Data represent the means ± SEM. The *P*-values were determined using paired Student’s *t*-test. ^**^P < 0.01.

**FIGURE 5:**
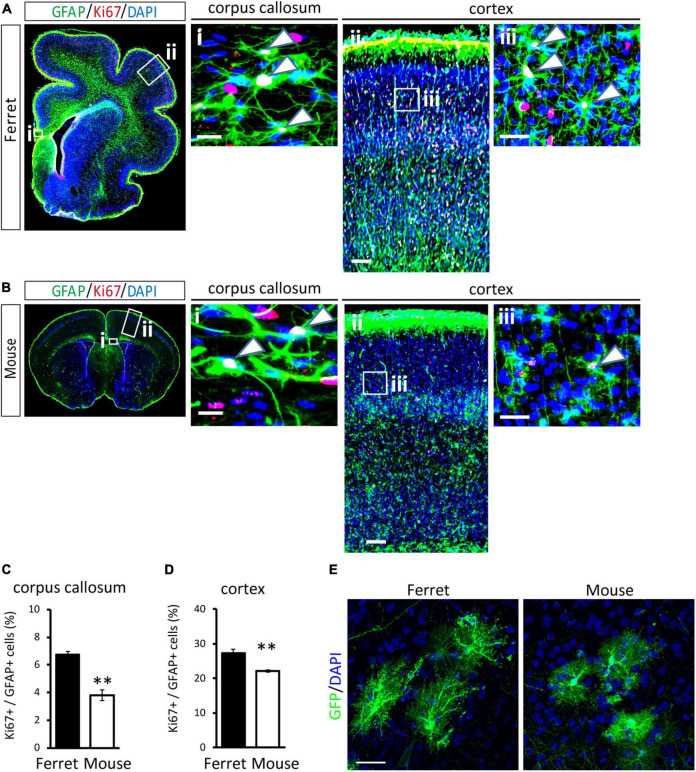
Proliferation of ferret and mouse astrocytes in the developing brain. **(A,B)** Representative images of GFAP and Ki67 in the cortex and corpus callosum of the postnatal ferret at P16 **(A)** and mouse at P8 **(B)**. Nuclei were stained with DAPI. Arrowheads indicate Ki67-positive astrocytes. **(C,D)** Proportion of Ki67-positive cells in GFAP-expressing astrocytes in the corpus callosum **(C)** and cortex **(D)** of ferret and mouse brains. **(E)** Representative images of GFP-transfected astrocytes in the cortex of the ferret (P36) and mouse (P30). Scale bars = 100 μm in **(A i)** and **(B i)**; 200 μm in **(A ii)** and **(B ii)**; 50 μm in **(A iii)** and **(B iii)**; and 30 μm in panel **(E)**. *n* = 4 animals each group. Data represent the means ± SEM. The *P*-values were determined using paired Student’s *t*-test. ^**^P < 0.01.

**FIGURE 6:**
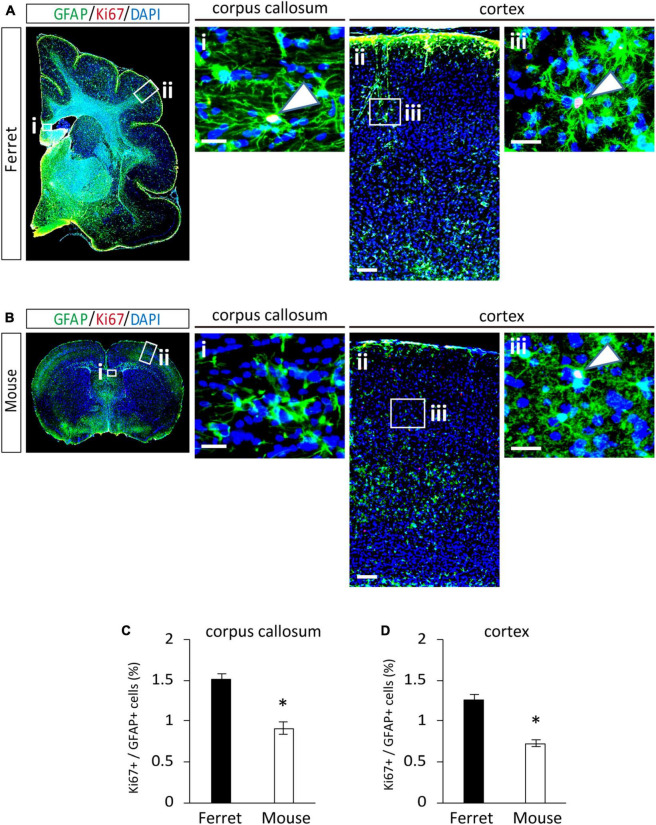
Proliferation of ferret and mouse astrocytes in the adult brain. **(A,B)** Representative images of GFAP and Ki67 in the cortex and corpus callosum of adult ferret at P100 **(A)** and mouse at P70 **(B)**. Nuclei were stained with DAPI (blue). Arrows indicate Ki67-positive astrocytes. **(C,D)** Proportion of Ki67-positive cells in GFAP-expressing astrocytes in the corpus callosum **(C)** and cortex **(D)** of ferret and mouse brains. Scale bars = 100 μm in **(A i)** and **(B i)**; 200 μm in **(A ii)** and **(B ii)**; and 50 μm in **(A iii)** and **(B iii)**. *n* = 4 animals each group. Data represent the means ± SEM. The *P*-values were determined using paired Student’s *t*-test. *P < 0.05.

To compare the proliferation ability of ferret and mouse astrocytes, we performed a BrdU assay and immunocytochemistry of Ki-67, a cell proliferation marker, in cultured cells. We observed a significantly higher rate of BrdU incorporation and Ki-67 positive cells in ferret astrocytes than in mouse astrocytes (Figures 4A–D). Immunohistochemical analysis of Ki-67 and GFAP antibodies in the postnatal and adult brains of ferrets and mice showed that the proliferation rate was higher in postnatal brains than in adult brains (Figures 5, 6). The proliferation rate was significantly higher in ferrets than in mice in the cortex and corpus callosum (Figures 5A–D). In adult brains, although the proliferation rate was low in both ferret and mouse brains, it was still higher in ferrets than in mice, both in the cortex and corpus callosum (Figures 6A–D). These results indicate that ferret astrocytes have a greater ability to proliferate compared to mouse astrocytes. In addition, the morphology of GFAP-positive astrocytes was consistent with that of cultured ferret astrocytes (Figure 1G). Ferret protoplasmic astrocytes had more branched and longer processes than mouse astrocytes in the postnatal (Figures 5Aiii,Biii) and adult brains (Figures 6Aiii,Biii). A similar trend was observed for ferret fibrous astrocytes in the corpus callosum (Figures 5Ai,Bi). To investigate detailed morphology of ferret and mouse astrocytes in the developing brain, we performed *in utero* electroporation of GFP-expressing vectors in the cortex during astrogenesis by using the *piggybac* system (Hamabe-Horiike et al., 2021). In consistent with a previous study of ferret visual cortex using fluorescent dyes (Lopez-Hidalgo et al., 2016), ferret protoplasmic astrocytes had larger cell size, longer primary processes in larger numbers, less spherical shapes, and more invaginations than mouse astrocytes (Figure 5E).

## Discussion

We investigated the features of ferret astrocytes using ferret and mouse astrocytic culture systems. Cultured ferret astrocytes had longer and branched processes and smaller cell bodies compared to mouse astrocytes. There were common genes in ferret and human astrocytes that were not expressed in mouse astrocytes. Ferret astrocytes showed a calcium response similar to that of human fetal astrocytes, along with a higher proliferation rate than that of mouse astrocytes *in vitro* and *in vivo*. These results suggest that the features of ferret astrocytes differ from those of mouse astrocytes and are common to those with human astrocytes.

We observed that cultured ferret astrocytes had long branched processes and small cell bodies (Figure 1). These features were similar to those of human astrocytes cultured without serum (Zhang et al., 2016). In the cortex, ferret protoplasmic astrocytes had larger cell size, more number of elongated primary processes (Figure 5E). These features are similar to those of human protoplasmic astrocytes. Human protoplasmic astrocytes had longer processes in larger numbers, larger cell diameters, and more overlap with adjacent cells than with mouse astrocytes (Vasile et al., 2017). These features may contribute to the coverage of more synapses and are considered to locally integrate information from a large number of cortical synapses (Vasile et al., 2017). However, we did not explore whether ferret astrocytes share their territories with other astrocytes in the cortex and corpus callosum, as observed in the human brain. Additionally, other types of astrocytes, such as interlaminar astroglia in cortical layer I and varicose projection astroglia in layers V–VI, are present in the human brain (Oberheim et al., 2009). Interestingly, these cells are also found in primates, although their roles in brain function remain largely unknown. Thus, further studies of the territorial overlap and types of astrocytes in the ferret brain are necessary to understand the functional roles of astrocytes in higher mammals. As a candidate gene responsible for morphological features, *Pmp2* may be involved in regulating ferret astrocyte size. We found that *Pmp2* was expressed in ferrets but not in mouse astrocytes (Table 2). Previous studies reported that PMP2 is expressed in humans but not in mouse astrocytes; infection of mouse astrocytes with a PMP2-expressing virus increased the cell diameter and number of primary processes (Kelley et al., 2018). Furthermore, PMP2 transports fatty acids to the cell membrane, contributing to lipid homeostasis in myelinating Schwann cells (Zenker et al., 2014). Because astrocytes contribute to the substantial fraction of lipids incorporated into the myelin of the central nervous system (Camargo et al., 2017), lipid metabolism in astrocytes may be enhanced to expand the white matter in higher mammals.

Cultured ferret astrocytes showed a calcium response to ATP but not to glutamate (Figures 3A,B), similar to human fetal astrocytes. Human fetal astrocytes do not react to glutamate, whereas mature astrocytes derived from the brain of people 8–68 years of age show elevated intracellular calcium levels in response to glutamate and ATP (Zhang et al., 2016). Accordingly, mature ferret astrocytes derived from post-astrogenesis stages (later than P14) may respond to glutamate. The application of glutamate to mature human astrocyte cultures induces a sharp and dose-dependent increase in intracellular Ca^2+^ levels by activating metabotropic glutamate receptor 5 (mGluR5) (Zhang et al., 2016). This phenomenon has also been observed in mouse astrocytes, and even in the neonatal brain (Sun et al., 2013). However, in our RNA-seq analysis, the mGluR5 expression levels in ferret astrocytes were similar to those in mouse astrocytes. mGluR1, another group I metabotropic glutamate receptor, showed lower expression in ferret astrocytes (data not shown), which may account for the differences in their calcium responses compared to in mouse cells. In addition, some mGluR5 expression levels in cultured astrocytes are different from those in the brain and affected by reactivity of the cells (Spampinato et al., 2018). Further *in vivo* study is needed to clarify the calcium response to ATP and glutamate in the ferret brain.

Consistent with the higher proliferation rate of cultured ferret astrocytes (Figure 4), ferret astrocytes, particularly in the postnatal cortex, showed higher proliferation than mouse astrocytes (Figures 5A,C). This higher proliferative ability of ferret astrocytes likely contributes to their high astrocyte counts and large brain size (Nedergaard et al., 2003). We prepared astrocyte cultures from P1 ferrets and P1–3 mouse cortices and observed that the astrocytes proliferated in P16 ferret brains and P8 mouse brains. These developmental stages in each species appear to correlate with the timing of astrogenesis. Astrogenesis occurs from E18 to around P7 in mice (Reemst et al., 2016), whereas in ferret it starts from E38 (4 days before birth) and finishes by approximately P14 (Gilardi and Kalebic, 2021). However, it is difficult to accurately compare the proliferation ability of astrocytes both *in vivo* and *in vitro*, as the period of astrogenesis in each species is not the same, and the proliferation rate of astrocytes changes during cortical development (Shoneye et al., 2020). In addition to proliferation ability, the duration of astrogenesis and brain development in ferrets is longer than that in the rodent brain (Barnette et al., 2009), which may also contribute to the generation of a large number of astrocytes in ferret brains.

Astrocytes have evolved to play important roles in brain function in higher animals. To understand their detailed roles in leading to higher cognitive functions with increased brain size, purified astrocytes from higher mammals must be analyzed. Isolation of ferret astrocytes using the McCarthy and de Vellis (1980) model was a good experimental tool for this study because of the ease of purification, large number of cells, low cost, efficient revival after freezing, and common features with human astrocytes. We found that *Calcoco2* and *Pmp2* were expressed in humans and ferrets but not in mouse astrocytes (Table 2). These molecules may be related to the specific roles of astrocytes in brain evolution in higher mammals. We observed high protein expression levels of PMP2 and CALCOCO2 in cultured ferret astrocytes (Supplementary Figure 2). PMP2 increases the size and processes of human astrocytes and is involved in lipid homeostasis in Schwann cells (Zenker et al., 2014; Kelley et al., 2018). In contrast, CALCOCO2 acts as an autophagic receptor for phosphorylated tau, facilitating its clearance and modulating inflammation by regulating nuclear factor-κB signaling downstream of the Toll-like receptor pathways (Till et al., 2013; Kim et al., 2014). Ferret astrocytes may exhibit different inflammatory responses under different pathological conditions (Li et al., 2021). Our culture system used serum-containing media and 3 weeks of cultivation were required to perform assays after harvesting the cortices. Serum exposure appears to alter astrocyte transcriptomes and morphology, resulting in fewer processes and hypertrophied cell bodies (Foo et al., 2011). Further gene expression analyses, cell proliferation assays, and calcium imaging studies on directly isolated astrocytes and in developing ferret brains are necessary to confirm our findings.

In conclusion, the characteristics of cultured ferret astrocytes differ from those of mouse astrocytes, such as their morphology, gene expression, calcium response, and proliferation ability, which are similar to the features of human astrocytes. Cultured ferret astrocytes may be useful for studying the roles of astrocytes in brain evolution, higher cognitive function, and dysfunction, such as in schizophrenia and autism, in higher mammals.

## Data availability statement

The data presented in the study are deposited in the GEO repository: https://www.ncbi.nlm.nih.gov/geo/, accession number GSE201199.

## Ethics statement

The animal study was reviewed and approved by the Animal Care and Use Committee of Kanazawa University (AP-183919).

## Author contributions

JR, TH, HK, and OH conceived and designed the experiments. JR, TH, DN, TM, and OH performed the experiments. JR, TH, HI, MT-I, TK, KH, TM, KS, YS, MM, and AT analyzed the data. TK, KH, TM, KS, YS, MM, AT, and HK contributed reagents, materials, and analysis tools. JR, TH, and OH wrote the manuscript. All authors contributed to the article and approved the submitted version.

## Footnotes

1
https://imagej.nih.gov/ij/

## Supplementary material

Supplementary Table 1 Gene expression data (in read count and FPKM) and gene lengths of all genes with identical gene symbols in ferret and mouse astrocytes.
