# Supplementary material for: Isolation of ferret astrocytes reveals their morphological, transcriptional, and functional differences from mouse astrocytes
Source: Front Cell Neurosci. 2022 Oct 6;16:877131. doi: 10.3389/fncel.2022.877131 (PMC9584309; doi:10.3389/fncel.2022.877131)
Supplement: Supplementary file 2 [file Table_2.DOCX]

**Supplementary Materials**


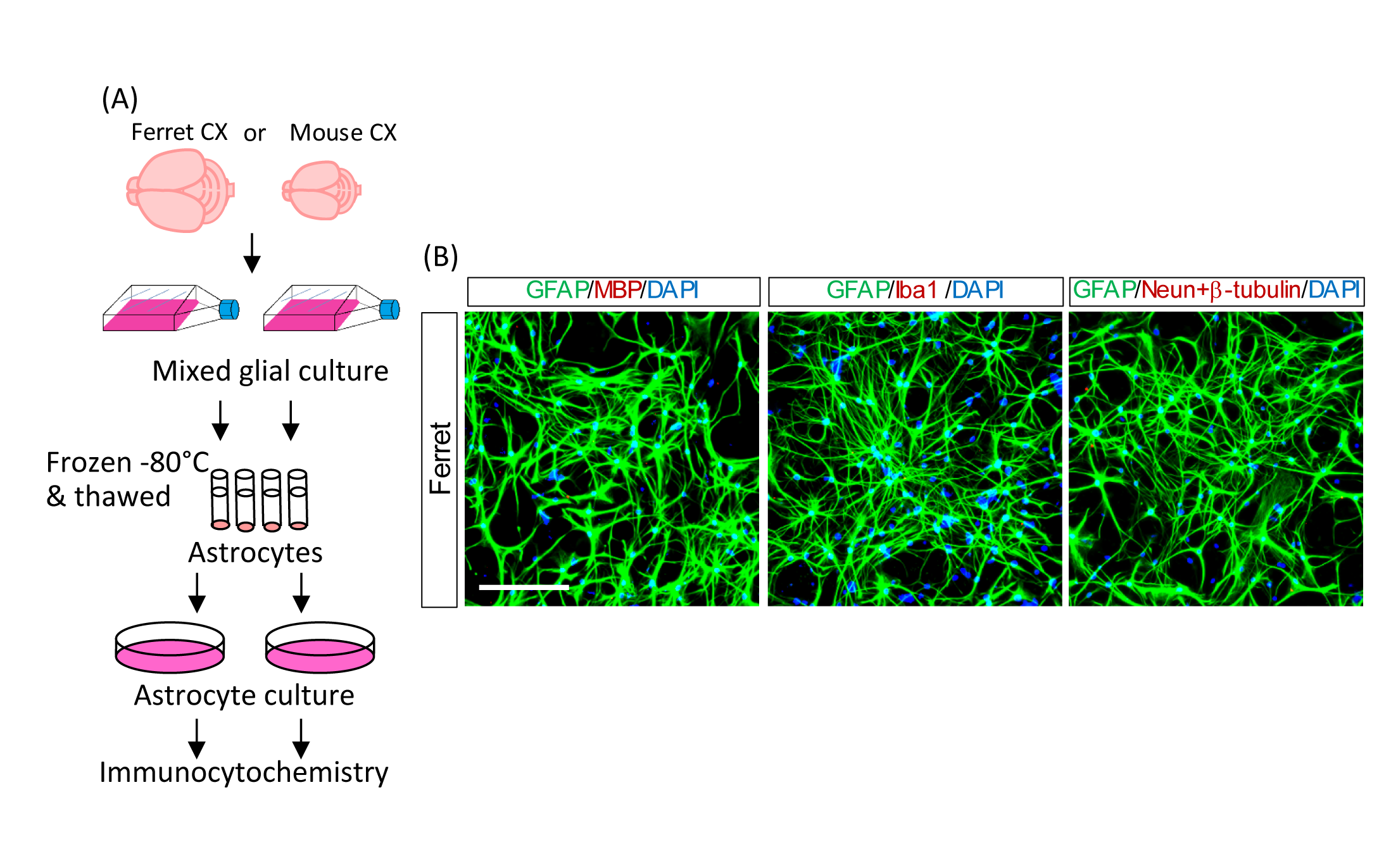


**Supplementary Figure 1 Ferret astrocyte culture after freeze and thaw cycle** (A) Experimental procedure of ferret astrocyte culture after freeze and thaw cycle. (B) Cultured ferret astrocytes after freezing and thawing were subjected to immunocytochemistry to detect GFAP, MBP, Iba1, NeuN, and β-III tubulin at 4 days after plating. Nuclei were counterstained with DAPI. Scale bar = 100 µm.


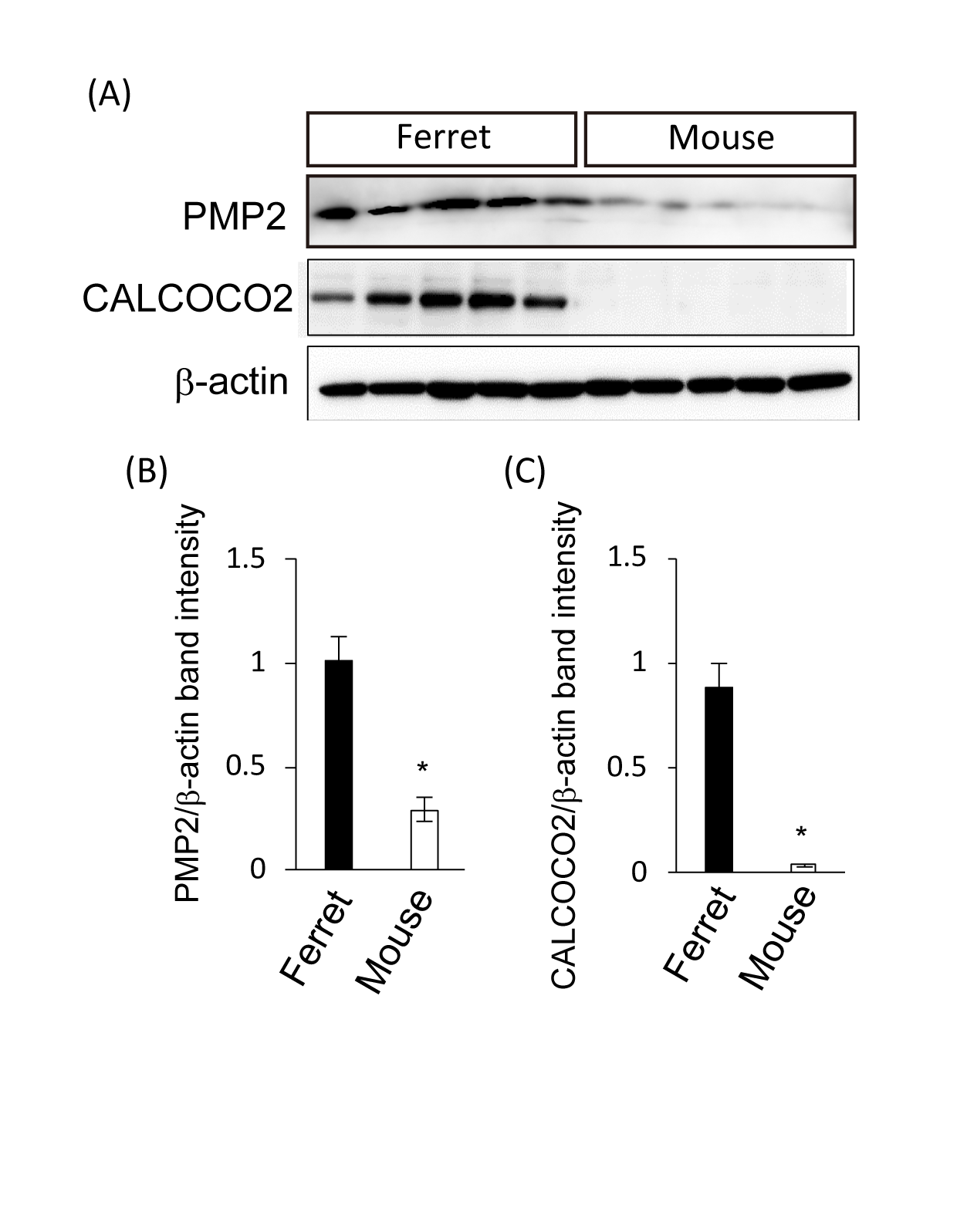


**Supplementary Figure 2 Protein expression of ferret astrocyte-enriched molecules** (A) Cultured astrocytes with serum were harvested at day 4 and subjected to western blot analyses with antibodies against PMP2 and CALCOCO2. (B and C) Relative optical density of PMP2 (B) and CALCOCO2 (C), normalized to the loading control β-actin, *n* = 5 independent cultures. Data represent the means ± SEM. The *P* values were determined using paired Student’s *t*-test. **P* < 0.05 between ferret and mouse astrocytes.
